# Unraveling a Lignocellulose-Decomposing Bacterial Consortium from Soil Associated with Dry Sugarcane Straw by Genomic-Centered Metagenomics

**DOI:** 10.3390/microorganisms9050995

**Published:** 2021-05-05

**Authors:** Bruno Weiss, Anna Carolina Oliveira Souza, Milena Tavares Lima Constancio, Danillo Oliveira Alvarenga, Victor S. Pylro, Lucia M. Carareto Alves, Alessandro M. Varani

**Affiliations:** 1 Departament of Technology, School of Agricultural and Veterinary Sciences, São Paulo State University (UNESP), Jaboticabal, São Paulo 14884-900, Brazil; bruno.weib@gmail.com (B.W.); annacarolinasouza@hotmail.com (A.C.O.S.); milena.tavares@hotmail.com (M.T.L.C.); danillo.alvarenga@gmail.com (D.O.A.); 2 Graduate Program in Agricultural and Livestock Microbiology, School of Agricultural and Veterinary Sciences, São Paulo State University (UNESP), Jaboticabal, São Paulo 14884-900, Brazil; 3 Microbial Ecology and Bioinformatics Laboratory, Department of Biology, Federal University of Lavras (UFLA), Lavras, Minas Gerais 37200-000, Brazil; victor.pylro@ufla.br

**Keywords:** lignocellulose, biotechnology, metabolic modeling, biofuels, community dynamics

## Abstract

Second-generation biofuel production is in high demand, but lignocellulosic biomass’ complexity impairs its use due to the vast diversity of enzymes necessary to execute the complete saccharification. In nature, lignocellulose can be rapidly deconstructed due to the division of biochemical labor effectuated in bacterial communities. Here, we analyzed the lignocellulolytic potential of a bacterial consortium obtained from soil and dry straw leftover from a sugarcane milling plant. This consortium was cultivated for 20 weeks in aerobic conditions using sugarcane bagasse as a sole carbon source. Scanning electron microscopy and chemical analyses registered modification of the sugarcane fiber’s appearance and biochemical composition, indicating that this consortium can deconstruct cellulose and hemicellulose but no lignin. A total of 52 metagenome-assembled genomes from eight bacterial classes (Actinobacteria, Alphaproteobacteria, Bacilli, Bacteroidia, Cytophagia, Gammaproteobacteria, Oligoflexia, and Thermoleophilia) were recovered from the consortium, in which ~46% of species showed no relevant modification in their abundance during the 20 weeks of cultivation, suggesting a mostly stable consortium. Their CAZymes repertoire indicated that many of the most abundant species are known to deconstruct lignin (e.g., *Chryseobacterium*) and carry sequences related to hemicellulose and cellulose deconstruction (e.g., *Chitinophaga*, *Niastella*, *Niabella*, and *Siphonobacter*). Taken together, our results unraveled the bacterial diversity, enzymatic potential, and effectiveness of this lignocellulose-decomposing bacterial consortium.

## 1. Introduction

The growing demand for renewable fuels to search for less environmentally impacting solutions has been nursing biofuel production improvements. Although first-generation sugarcane ethanol has a relatively high yield, it can also generate large amounts of lignocellulosic biomass or bagasse residues. This remaining biomass contains more than 65% of the plants’ fixed energy and is organized systematically through various polymers [1]. Lignocellulose can be partially deconstructed into fermentable sugars, increasing the overall fuel yield by its use in second-generation ethanol production [2,3,4]. Nevertheless, lignocellulosic biomass deconstruction still is a challenging process.

The polymers found in the lignocellulosic biomass are mostly cellulose, hemicellulose, pectin, and lignin [5]. Cellulose, the most abundant organic polymer on Earth, is a linear polysaccharide composed of glucose units, forming long chains in a rich intermolecular interaction web, which results in a very rigid, crystalline, or quasi-crystalline structure alternating with fewer crystalline regions [6,7]. Conversely, hemicellulose has an amorphous and overall flexible structure, more amenable to deconstruction [8]. Pectin, an essential component of plant tissues, such as fruits, is usually absent or present in minimal amounts in mature sugarcane tissues [9]. Lignin, the second most abundant organic polymer on Earth, acts as a retardant to cellulose and hemicellulose breakdown [10]. However, the sugarcane bagasse contains lower lignin amounts, accounting for 18% of the sugarcane biomass [11]. Lignin is a highly branched, phenolic polymer composed of diverse monomers, and so its decomposition does not result in fermentative sugars [12]. Various enzymes found in different bacterial phylums are necessary for lignocellulose deconstruction, some of which are grouped under glycosyl hydrolases (GHs) families by the Enzyme Commission Number (EC number) classification schema. For instance, cellulose and hemicellulose can be degraded aerobically or anaerobically mainly by Actinobacteria and Firmicutes phylums [6]. Conversely, the depolymerization of lignin can be done by lignin-oxidizing enzymes produced from soil filamentous prokaryotes (for a review, please see [6]).

Bacterial communities or consortia can efficiently deconstruct lignocellulose in nature [13,14] by synthesizing enzymes, such as GHs, that act synergistically to breakdown the biomass [15,16]. The hydrolysis of biomass polysaccharides reduces sugars, particularly monosaccharides as glucose and xylose, which may serve as a carbon source for the community. Interestingly, cellulases and hemicellulases can also be found free or as multimeric complexes called cellulosomes, including carbohydrate-binding modules (CBMs) that bind to the substrate surface, increasing the hydrolytic efficiency [17]. These CBMs are commonly produced by fungi and bacteria consortiums. Another plethora of lignocellulosic deconstruction-related enzymes that are produced by bacterial consortia is grouped into the glycosyl transferases (GTs), auxiliary activity (AA), and polysaccharide lyases (PLs) families.

Here, we studied lignocellulose-deconstructing bacterial consortium from surface soil and above-ground sugarcane straw, previously characterized by our research group [18] using the metagenomics approach. This bacterial consortium was cultivated and enriched in vitro for 20 weeks using lignocellulosic biomass (sugarcane bagasse) as the only carbon source. Two contrasting time samplings of cultivation (2nd and 20th weeks) were analyzed to evaluate the consortium’s ability to deconstruct and use sugarcane bagasse as a carbon source. We have also employed a method to identify the bacterial community closely associated with the sugarcane bagasse fibers (attached-fraction) and non-closely associated with the sugarcane bagasse fibers (free-fraction).

Finally, we propose a model of taxonomy-defined Division of Biochemical Labor (DoBL) for this consortium. Here, we define DoBL as the concatenation of syntrophic relationships between a bacterial consortium’s composing members, observing each step in executing a defined complex biochemical reaction designated to one or a few members (species or groups) of such community. Our results clarify the metabolic process involved in lignocellulose deconstruction and the relationship between this system’s parts, contributing to future improvements in using this bacterial consortium and technology in biofuels production.

## 2. Materials and Methods

### 2.1. Sampling of Lignocellulose-Deconstructing Bacterial Consortium

The bacterial consortia were obtained by sampling a sugarcane plantation cultivation soil (21°19′23.5″ S 48°09′12.3″ W, elevation: 534.1 m), following the methods described by [18]. For enrichment, a 500 μL supernatant aliquot was inoculated in a sterile Bushnell Haas broth (BHB) medium consisting (g·L^−1^) of magnesium sulfate, 0.2; calcium chloride, 0.02; monopotassium phosphate (monobasic), 1.0; ammonium phosphate, 0.02; potassium nitrate, 1.0; ferric chloride, 0.05; containing 2.0 mg/mL of washed, dried, crushed, and sieved (32 mesh) sugarcane bagasse as the only carbon source.

### 2.2. Adaptation of the Bacterial Consortium to the Culture Medium

**Figure 1:**
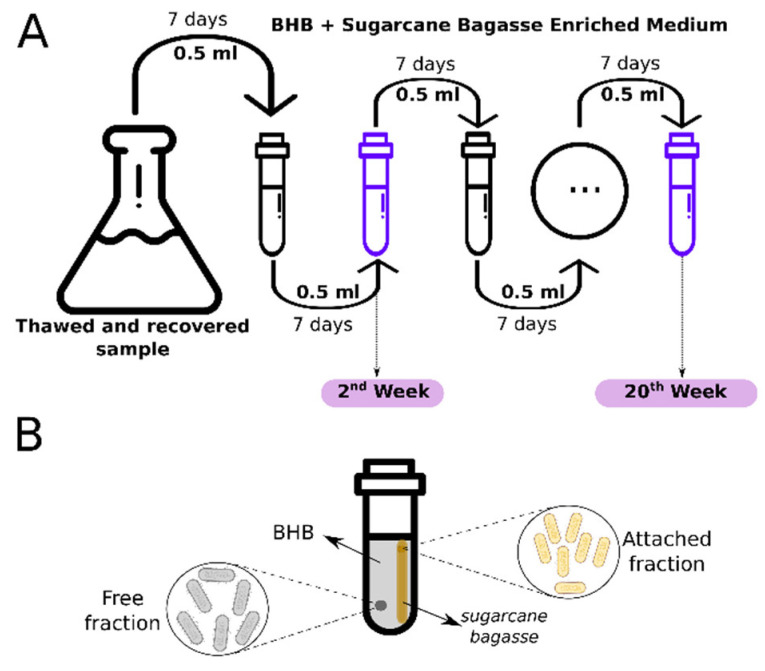
Detailing of the methodology from the adaptation of the consortium and the time samples, 2nd and the 20th weeks of consortium cultivation (**A**), and the process to recover bacteria that were closely associated (attached-fraction) and non-closely associated (free-fraction) to the sugarcane bagasse fibers (**B**).

The bacterial consortium’s adaptation process to the medium containing milled sugarcane bagasse was conducted as follows: the culture suspension was incubated for seven days at 30 °C, with constant shaking at 150 rpm. After this period, a 500 μL aliquot was transferred to a new enriched medium containing 50 mL of BHB + sugarcane bagasse. This procedure was repeated for 25 weeks, and for each week, several aliquots of 800 μL were stocked in glycerol at −80 °C. For the experimental purpose (Figure 1A), six thawed samples from the first week of cultivation were recovered; three were cultivated for 7 days (30 °C, 150 rpm) for the experiments from the second week. The other three samples were cultivated, under the same conditions, through 20 weeks, with weekly transfer to a new media (BHB + sugarcane bagasse).

### 2.3. Metagenomic DNA Extraction and Sequencing

We adopted two contrasting time samples, the 2nd and the 20th weeks of consortium cultivation, for the metagenomic study. We recovered the bacteria that were closely associated (attached-fraction) and non-closely associated (free-fraction) to the sugarcane bagasse fibers from the medium containing 50 mL of BHB + sugarcane bagasse (Figure 1B).

The attached and free fractions of the culture were separated by filtration on Whatman No. 1 filter paper using sterile material, following procedures described in [19]. The supernatant from this step was discarded, and the solid part was carried out to the DNA extraction step. For each week of bacterial consortia cultivation (2nd and the 20th week), 1 mL was used for DNA extraction, and 800 μL stocked in glycerol at −80 °C.

Total DNA was extracted using the Wizard^®^ Genomic DNA Purification Kit (Promega Corporation, Madison, WI, USA), following the manufacturer’s instructions. The purity of the extracted DNA was checked with the Nanodrop ND-1000 spectrophotometer (Nanodrop Technologies, Wilmington, DE, USA) (260/280 nm ratio) and quantified by Qubit^®^ fluorometer using the dsDNA BR Assay Kit (Thermo Fischer Scientific, Waltham, MA, USA) according to the instructions of the manufacturer. The DNA integrity was confirmed by electrophoresis in a 0.8% agarose gel with 1 × TBE buffer.

Total metagenome DNA libraries were prepared with Illumina Nextera DNA Library Prep Kit (Illumina, San Diego, CA, USA). Sequencing was performed at Illumina^®^ HiSeq 2500, on a Flow Cell v. 4, using HiSeq SBS v. 4 kits (Illumina, San Diego, CA, USA), 2 × 100 bp paired-end reads. Read quality was evaluated with FastQC 0.11.4, and the removal of adapters was performed with Trimmomatic v. 0.36 [20]. Reads shorter than 50 bp and with PHRED values below 23 were filtered out from further analysis.

### 2.4. Scanning Electron Microscopy of Sugarcane Bagasse Fibers

Four different circumstances were compared: only the fiber-rich sterile medium alone (i) and kept under shaking (150 rpm) (ii), and the fiber-rich medium with the community and kept under shaking (150 rpm) during the 2nd (iii) and 20th (iv) weeks of cultivation. Both samples were thawed and recovered, then cultivated in BHB medium with sugarcane bagasse for five days. These samples were filtered and separated in the attached and free fractions. Post-fixation was performed with osmium tetroxide (OsO_4_) and ethanol dehydration (99 to 10%). The fixed samples were then assembled over the support and coated with gold (20–30 mm). Two sterile mediums were used (30 °C, 150 rpm, and only at 30 °C) for negative control. Imaging was performed under the scanning electron microscope (Joel JSM6610LV) in a range of magnification from 1000× to 5000×.

### 2.5. Evaluation of the Decomposition of Lignocellulosic Biomass

The analysis of cellulose, hemicellulose, and lignin in sugarcane bagasse fibrous fractions was performed using the Ankom filter bag technique and an Automated Fiber Analyzer (ANKOM Technology, Macedon, New York, NY, USA). The 2nd and 20th weeks only from the sugarcane bagasse fibers were analyzed. The neutral detergent fiber (NDF), acid detergent fiber (ADF), and lignin content were measured using procedures described by [21].

For the NDF analyses, 100 mL of neutral detergent solution (30.0 g sodium sulfate + 10.0 mg ethylene glycol + 18.0 g EDTA + 6.81 g sodium borate + 4.56 g sodium phosphate) were added to 1 L of distilled water with the biomass. For the ADF analyses, 100 mL of acid detergent solution (28.5 mL sulfuric acid + 20.0 g of cetyltrimethylammonium bromide (CTAB)) was added to the biomass. The biomass samples were previously weighed for both analyses, followed by boiling for one hour in a fiber digester (MA- 455 Marconi^®^). The samples were vacuum filtered and washed three times with distilled hot water and washed two times with pure acetone under ambient temperature. The samples were transferred to a kiln under 105 °C and weighed. All analyses were evaluated in six replicates for the 2nd and 20th weeks of cultivation.

### 2.6. High-Performance Liquid Chromatography and Sugar Yields in Culture Medium

Fifteen milliliters of sample supernatants from the 2nd and 20th weeks of cultivation were collected by centrifugation (Sorvall centrifuge at 16,266× *g* for 96 min at 4 °C) and concentrated (Eppendorf AG 22331 Hamburg Concentrator Plus) to a final volume of 1 mL. All samples were filtered through a 0.45 μM cellulose ester filter and further analyzed by liquid chromatography on a high-performance liquid chromatography (HPLC) system equipped with a refractive index detector (RID) (Shimadzu, (Kyoto, Japan) model 100 RID-10A). Sugar separation was performed by a Supelcosil LC-NH2 column (25 cm × 4.6 mm) with a constant flow rate of 1 mL·min^−1^ using acetonitrile: H2O buffer (75:25, *v:v*) at 35 °C. The sugar yields hydrolysis of lignocellulosic biomass was calculated according to [22]. All analyses were evaluated in triplicates.

### 2.7. Metagenomic Binning, Quality Assessment, and Taxonomy Assignment

A genome-centric approach was applied to evaluate the bacterial consortium composition, and the abundance of metagenome-assembled genomes (MAGs) was obtained through MetaWRAP v. 1.2.2 pipeline using standard parameters [23]. Megahit v1.0.6-gfb1e59b [24] was used for the metagenome assembly. The binned genomes’ completeness and contamination were estimated using CheckM v.1.4.0 [25]. GTDBtk v. 0.3.0 [26], together with Kraken2 v. 2.0.8 [27] using the complete GenBank RefSeq Database [28] were used to MAGs taxonomy assignment.

### 2.8. Functional, Metabolic Pathways and Carbohydrate Hydrolases Annotation and Analysis

Each MAG was annotated using the RAST server [29] and KEGG GhostKOALA [30]. The dbCAN2 meta server [31] and EggNOG v. 5.0 database [32] were used to search for carbohydrate-active domains in each identified gene. Metabolic modeling of the consortia was done using the EnrichM pipeline (https://github.com/geronimp/enrichM (accessed on 10 March 2021)). The CAZY enzyme heatmap figures were made with the MetabolisHMM tool [33].

### 2.9. Phylogenetic Analysis of the Identified MAGs

A phylogenetic tree was reconstructed using the maximum-likelihood approach, based on MAGs shared genes identified with Roary v3.13 [34] tool and considering 60% identity and 80% of similarity. The sequences were aligned using MAFFT v7.453 [35]. The best-fit models for each alignment were calculated, and maximum likelihood analyses were performed using RaXML v8.2.12 [36], with 1000 bootstrap resampling.

## 3. Results

### 3.1. Scanning Electron Microscopy Suggests the Role of the Consortium in the Deconstruction of Lignocellulose Biomass

**Figure 2:**
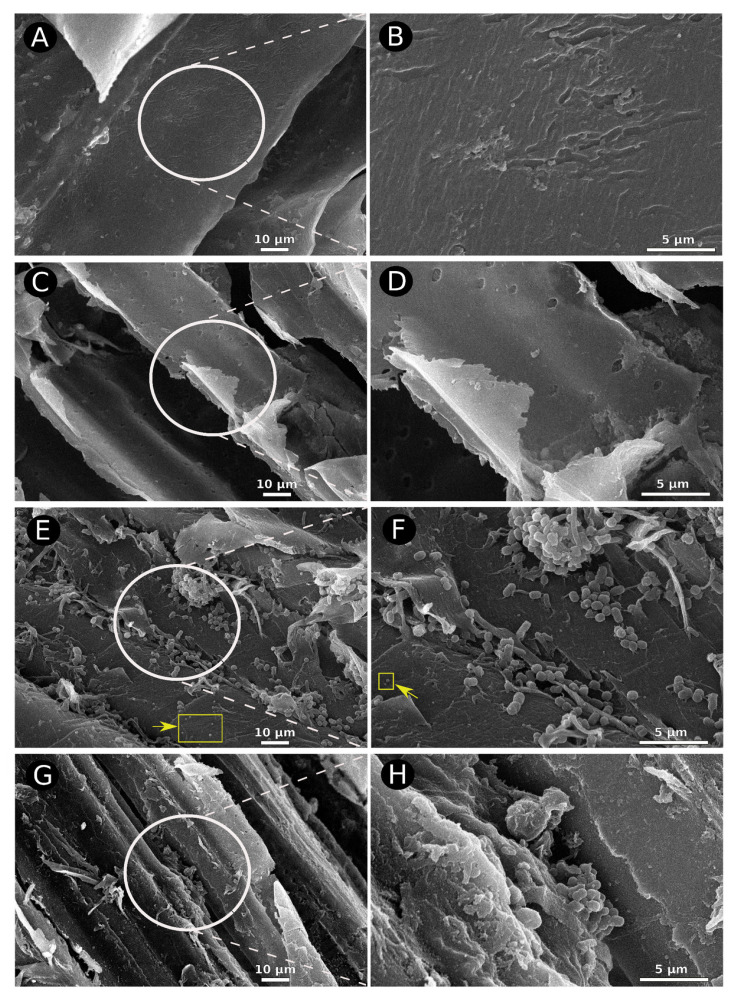
Scanning electron microscopy of sugarcane fibers used as a sole carbon source in bacterial culture media. (**A**,**B**) show the sugarcane fibers in sterile culture. (**C**,**D**) show the sugarcane fibers in agitated and sterile culture for ten days. (**E**,**F**) show the sugarcane fibers in a culture media from the 2nd week consortium growing under agitation, and the yellow arrows indicate structures resembling pseudo-lignin droplets. (**G**,**H**) show the sugarcane fibers in a culture media from the 20th week consortium growing under agitation. (**A**,**C**,**E**,**G**) show regions under 1000× amplification, while (**B**,**D**,**F**,**H**) are insets of (**A**,**C**,**E**,**G**) respectively, showing 5000× amplification.

A flat and compact structure was observed without bagasse fiber peels, indicating that the autoclaving process did not interfere with the sugarcane bagasse fiber structure (Figure 2A,B). The material showed signs of peeling when kept ten days in mechanical agitation in the sterile cultivation medium. However, it still kept a compact structure and slight peeling (Figure 2C,D). Figure 2E–H showed an evident alteration in the consortiums’ fibers’ structure and colonization, causing flaking, peeling, and overall physical deconstruction of the sugarcane fibers. The 2nd and 20th weeks of cultivation (Figure 2E–H) showed a deconstruction of the planar and compact structure of the bagasse, the presence of cracks and peeling, and the adhesion of various bacterial types on their surface.

Moreover, it was also possible to visualize some structures resembling pseudo-lignin droplets formed from the condensation of sugar degradation and lignin fragments, mainly on the 2nd week of cultivation (yellow arrows on Figure 2E,F and Figure S1). Therefore, these pseudo-lignin droplets might correspond to the degradation of the biomass itself. Overall, these findings indicate that the structure of sugarcane bagasse was modified by cultivation with the consortium, leading to partial fiber disruption, exposing the fibers, and facilitating bacteria’s adhesion to hydrolyze the lignocellulosic fractions. Interestingly, distinct bacterial morphological types are observed attached to the sugarcane fibers, suggesting that lignocellulosic deconstruction occurred through different microorganisms. These results strongly suggest that the bacterial consortium might be changing the lignocellulose fiber structure to use it as a carbon source.

### 3.2. Polysaccharide and Glucose Quantification Indicates a Dynamic Process of Lignocellulosic Biomass Deconstruction

**Figure 3:**
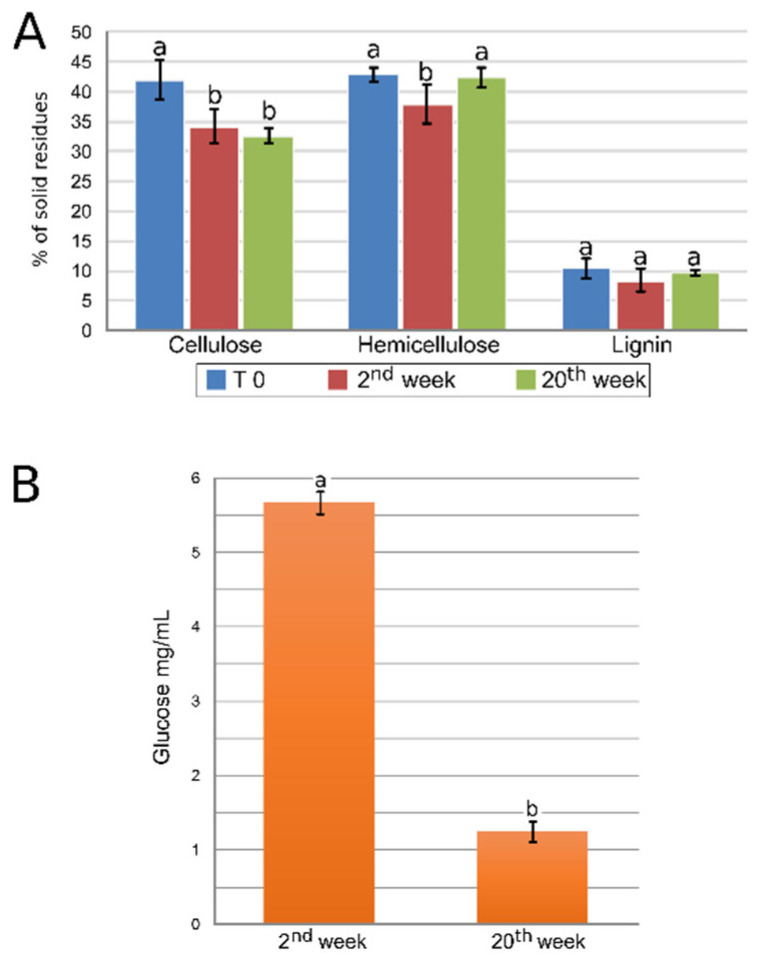
Quantities of cellulose, hemicellulose, lignin, and glucose in sugarcane bagasse after cultivation with a bacterial consortium. (**A**). Cellulose, hemicellulose, and lignin assayed before (T 0) and during the 2nd and the 20th week of cultivation. Error bars indicate the standard error of six independent biological replicates. (**B**). Quantity of glucose assayed at the 2nd and the 20th week of cultivation. Error bars indicate three independent biological replicates’ standard error. The data were statistically analyzed using Tukey’s test at 1% probability (*p* < 0.01). “a” and “b” indicate significant statistical difference among samples.

Estimates of the decomposition of lignocellulosic biomass and glucose consumption indicated that the bacterial consortia could degrade cellulose and hemicellulose but not lignin (Figure 3A). The deconstruction of cellulose, but not hemicellulose, was observed during the 20th week. We also observed that glucose availability during the 2nd week of cultivation was approximately 3.5× higher than in the 20th week (Figure 3B), following the pseudo-lignin droplets’ visualization on Figure 2E,F and, thus, indicating that the degradation of cellulose is occurring. Moreover, hydrolysis efficiency analysis showed a glucose yield of 75.6% during the 2nd week and negative values (−36.7%) during the 20th week of cultivation (Table S1). These results indicate a dynamic lignocellulosic decomposition process, suggesting that the consortium released more glucose than it consumed during the 2nd week and consumed almost all glucose released in the 20th week.

### 3.3. Metagenome Characterization Uncovered Four Main Bacterial phyla in the Lignocellulolytic Community

A total of ~360 Gb of high-quality paired-end reads were generated and assembled for each week of cultivation and respective fractions (free and attached fractions) (Table S2). In general, each sample was assembled into ~200 Mb and contained in more than 130,000 scaffolds (>300 bp), showing an average N50 of ~5 kb. The average nucleotide identity (ANI) between the fractions (free and attached) across the 2nd and 20th weeks is on average 98%, corroborating a near-identical taxonomic composition between all the samples.

**Figure 4:**
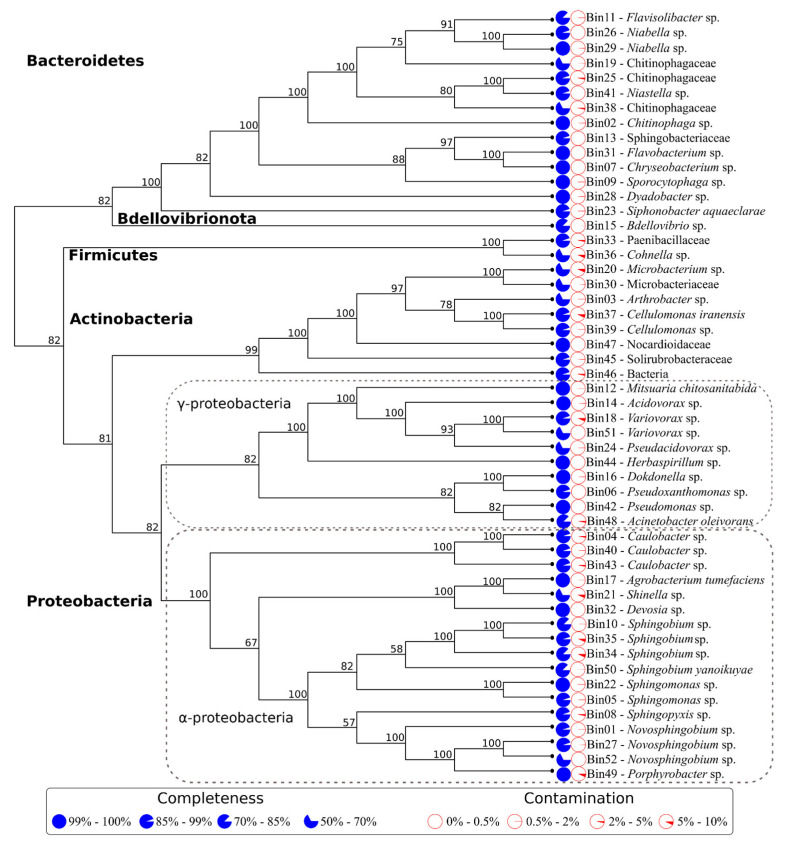
Multilocus maximum-likelihood tree showing the metagenome-assembled genomes (MAGs) diversity found in the consortium (as total binned metagenome). Names on the branch tips are followed by a blue circle indicating the completeness level and a red circle indicating the contamination level estimated by CheckM (more details for each binned sequence are presented in Table S3).

Considering the high similarity between the 2nd the 20th weeks’ assemblages, the trimmed reads from all samples were pooled together to improve the quality of each MAG obtained and assembled into ~374 Mb with an average GC content of 60% (Table S2). We recovered a total of 52 metagenome-assembled genomes (MAGs), resulting in 240 Mb of total genomic attribution of the metagenomic assembly (mean of 4.63 Mpb for each MAG) (Figure 4). The unbinned sequences (~130Mb) were mainly related to low-quality bins (completeness below 50% and contamination above 20%) and eukaryotic contamination (mainly derived from sugarcane fibers). Moreover, functional predictions reported incomplete and non-essential pathways related to biomass deconstruction among the unbinned sequences, and thus, they were not considered for further analyses.

The MAGs were taxonomically assigned to four main phyla (Actinobacteria [*n* = 8], Bacteroidetes [*n* = 14], Firmicutes [*n* = 2], and Proteobacteria [*n* = 28]), eight classes (Actinobacteria [*n* = 7], Alphaproteobacteria [*n* = 17], Bacilli [*n* = 2], Bacteroidia [*n* = 11], Cytophagia [*n* = 3], Gammaproteobacteria [*n* = 10], Oligoflexia [*n* = 1], and Thermoleophilia [*n* = 1] (Figure 4 and Table S3).

Four of the 52 MAGs showed an estimated 100% completeness and less than 5% contamination, including *Chryseobacterium* sp. Bin7, which showed no contamination. Considering the criteria established by [25], we found that 31 (59.6%) of the 52 MAGs in the consortium could be classified as near-complete (over 90% complete) and 16 (30.6%) as substantially complete (between 70 and 90% complete).

### 3.4. Species Relative Abundance Changes Indicate a Dynamic Community Deconstructing the Lignocellulosic Biomass

**Figure 5:**
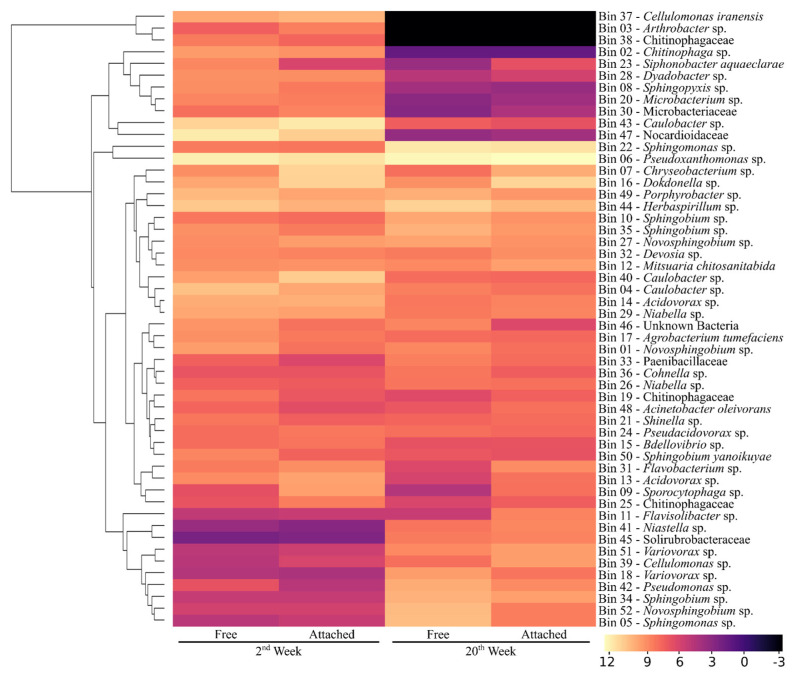
Global abundance heatmap and hierarchical clustering of each MAGs/Bins across the 2nd and 20th weeks of cultivation and their associated fractions (free and attached). Lighter colors indicate higher relative abundance concerning the metagenome.

The relative abundance of each MAG based on the reads mapping assignment showed some differences between the free and attached fractions along the cultivated weeks, but most MAGs were found in both fractions (Figure 5). For instance, three MAGs found in the 2nd week in both fractions were not observed in both fractions of the 20th week of cultivation: *Arthrobacter* sp. Bin 3, *Cellulomonas iranensis* Bin 37, and Chitinophagaceae Bin 38.

Furthermore, a relative abundance reduction between the 2nd and the 20th week was drastic for *Caulobacter* sp. Bin43 and Nocardioidaceae Bin47. Reduction in abundance was observable but less intense in *Chryseobacterium* sp. Bin7, *Caulobacter* sp. Bin40, *Caulobacter* sp. Bin4, *Acidovorax* sp. Bin 14, *Niabella* sp. Bin29, *Dokdonella* sp. Bin16, *Acidovorax* sp. Bin13, *Chitinophaga* sp. Bin2, *Porphyrobacter* sp. Bin49, *Dyadobacter* sp. Bin28, *Sporocytophaga* sp. Bin9, *Sphingopyxis* sp. Bin8, and *Microbacterium* sp. Bin20, respectively, in decreasing order.

An increase in relative abundance between the 2nd and the 20th week was conspicuous to *Pseudoxanthomonas* sp. Bin6 and *Sphingomonas* sp. Bin22. Some increase was also observed in *Sphingomonas* sp. Bin5, *Novosphingobium* sp. Bin52, *Sphingobium* sp. Bin34, *Herbaspirillum* sp. Bin 44, *Pseudomonas* sp. Bin42, *Sphingobium* sp. Bin35, *Sphingobium* sp. Bin 10, *Variovorax* sp. Bin51, *Variovorax* sp. Bin18, *Cellulomonas* sp. Bin39, and Solirubrobacteraceae Bin45, respectively, in decreasing order. The other 24 MAGs with species assignments found in these communities showed no considerable change in relative abundance between the 2nd and 20th weeks of sampling.

### 3.5. CAZY Enzymes Abundance and Distribution Indicates a Synergistic Action of Each MAG to Degrade the Lignocellulosic Mass

**Figure 6:**
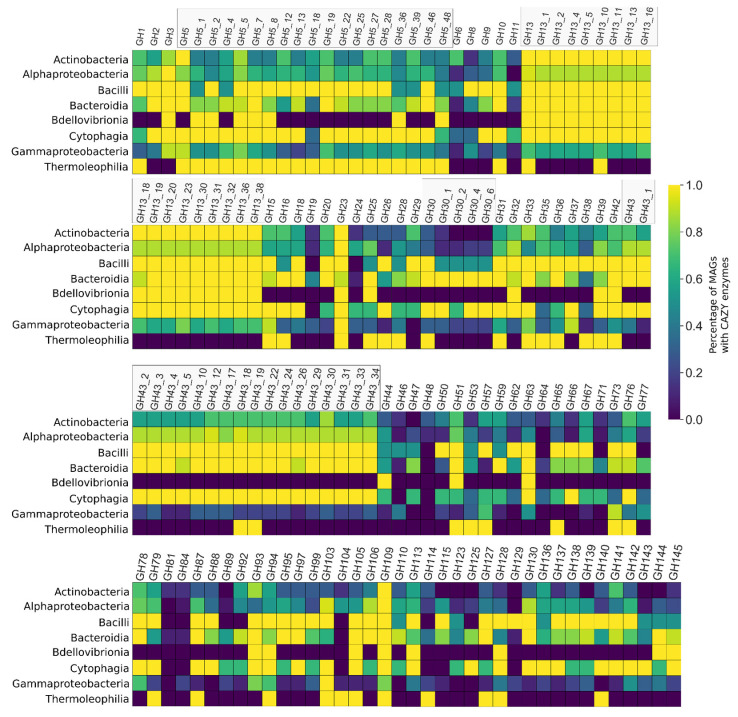
Heatmap showing the abundance of glycosyl hydrolases (GHs) identified in the consortium metagenome in proportion to each category and each taxonomic class.

**Figure 7:**
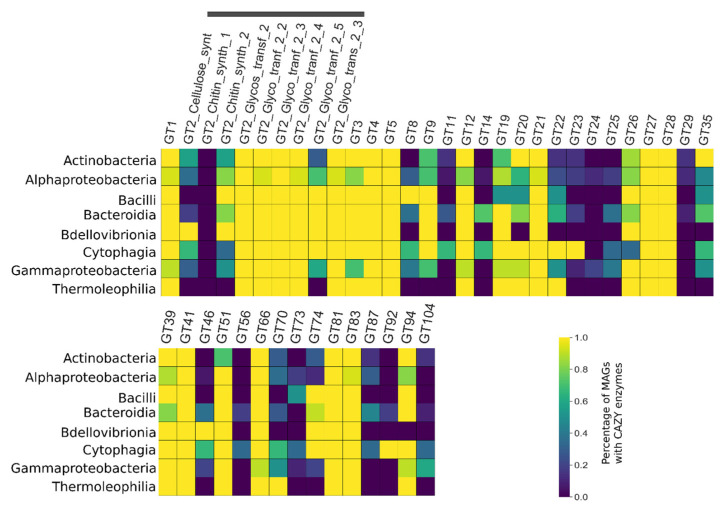
Heatmap showing the abundance of glycosyl transferases (GT) identified in the consortia metagenome in proportion to the total of each category and each taxonomic class.

**Figure 8:**
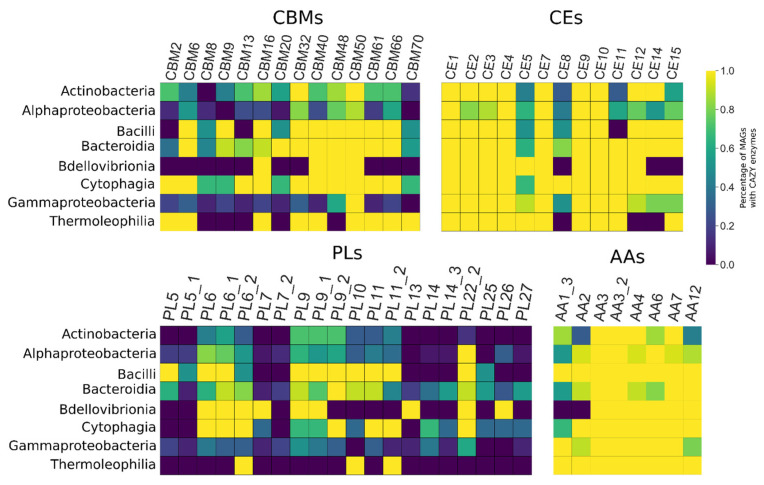
Heatmap showing the abundance of carbohydrate-binding modules (CBMs), carbohydrate esterase (CEs), polysaccharide lyase (PLs), and auxiliary activities (AAs) identified in the consortia metagenome in proportion to the total of each category and each taxonomic class.

At least 236 different CAZY enzymes families or subfamilies totaling at least 41,450 domains with the potential to participate in the deconstruction of the lignocellulosic biomass were identified in the consortium metagenome (8 AAs, 14 CBMs, 13 CEs, 35 GTs, and 146 GHs) (Figure 6, Figure 7 and Figure 8, and Table S4).

**Figure 9:**
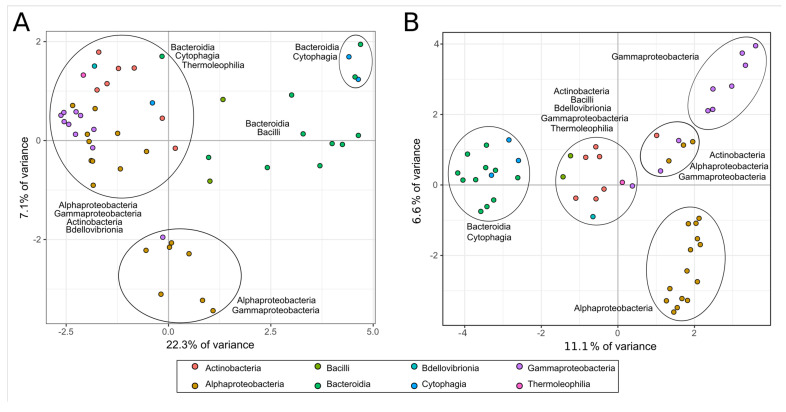
Principal component analyses showing the relationship between taxonomy (colors) and variation in the number of sequences identified as indicative of lignocellulose deconstruction. (**A**). CAZymes, (**B**). KEGG EC-numbers.

Principal component analyses (PCAs) evaluating the quantitative relationship between the number of sequences related to the deconstruction of lignocellulose and taxonomy indicated differentiation between taxonomic groups and the number of CAZyme sequences and taxonomic groups, and the number of KEGG EC-number sequences (Figure 9A,B). Classes Alphaproteobacteria, Gammaproteobacteria, and Oligoflexia tend to group (all belonging to phylum Proteobacteria) in both PCAs. In the same fashion, classes Cytophagia and Bacteroidia (belonging to phylum Bacteroides) tend to group, while separated from Proteobacteria groups—and it is also the case for Actinobacteria and Thermoleophilia classes (phylum Actinobacteria). The relationship between the number of KEGG EC-number and taxonomy results in more clearly defined groups than the relationship between the CAZyme sequences and taxonomy.

**Figure 10:**
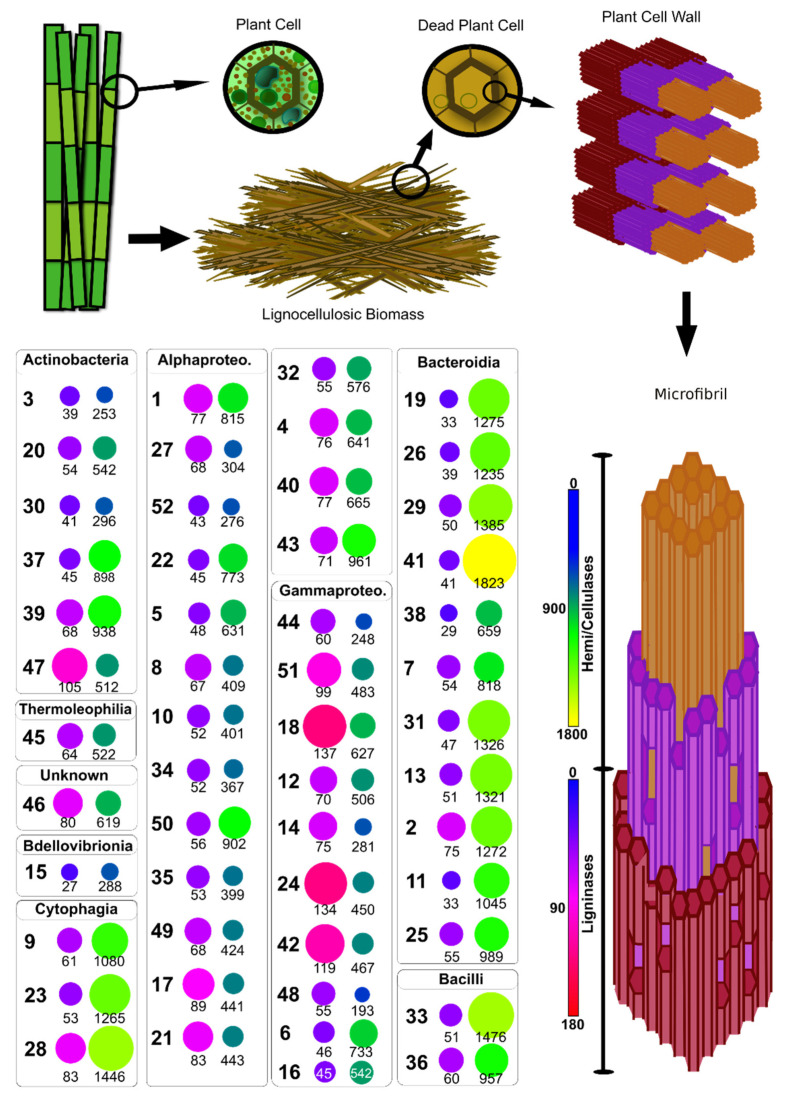
Model of the potential participation of each bin of the consortium’s metagenome in the process of the deconstruction of lignocellulosic biomass. Bins with higher amounts of sequences related to this process are considered more relevant to the process. The MAGs were grouped depending on their Class. Circles on the left show ligninases, circles on the right show hemicellulases and cellulases together.

The pooling of all the CAZyme families into the ligninases group and hemicellulases and cellulases group showed differences in each class’ relevance found in the total metagenome. This finding suggests further participation of each species in the consortium on the DoBL related to the deconstruction of lignocellulose, as it is evident in the model of metabolic potential proposed (Figure 10). The central premise adopted in the metabolic model proposed is that the quantity of sequences related to each polymer’s deconstruction found in the bagasse is proportional to the species’ relevance to the effectuation of such reaction in the process of deconstructing lignocellulose—i.e., more sequences, more relevance.

Bacteroidia, Cytophagia (both phylum Bacteroides), and Bacilli (phylum Firmicutes) are the classes in which we can find most MAGs with the higher potential to deconstruct hemicellulose and cellulose. For instance, *Niastella* sp. Bin41 (Bacteroidia) shows notably high potential to deconstruct hemicellulose and cellulose, offering more than 1800 domain sequences linked to this process, followed by Paenibacillaceae Bin33 (Bacilli, 1476 sequences) and *Dyadobacter* sp. Bin28 (Cytophagia, 1446 sequences). *Niabella* sp. Bin29 (Bacteroidia), *Flavobacterium* sp. Bin31 (Bacteroidia), Sphingobacteriaceae Bin13 (Bacteroidia), Chitinophagaceae Bin19 (Bacteroidia), *Chitinophaga* sp. Bin2 (Bacteroidia), *Niabella* sp. Bin26 (Bacteroidia), and *Siphonobacter* sp. Bin23 (Cytophagia) shows more than 1200 domain sequences related to hemi/cellulases, representing its relevance to this process (Figure 10).

Gammaproteobacteria is the class with most MAGs showing higher potential to deconstruct lignin. The three MAGs with higher potential in the consortium belong to this class: *Variovorax* sp. Bin18, *Pseudoacidovorax* sp. Bin24, and *Pseudomonas* sp. Bin42, all of which presented more than 115 domain sequences. Additionally, in this class, *Variovorax* sp. Bin51 showed 99 domain sequences. In class Actinobacteria, Nocardioidaceae Bin47 showed notably high potential, reaching 105 domain sequences. Only four more MAGs showed more than 80 domain sequences, namely, *Agrobacterium tumefaciens* Bin17 (Alphaproteobacteria, 89 sequences), *Shinella* sp. Bin21 (Alphaproteobacteria, 83 sequences), *Dyadobacter* sp. Bin28 (Cytophagia, 83 sequences), and Unknown Bin46 (Unknown, 80 sequences) (Figure 10).

## 4. Discussion

This work characterized a bacterial consortium related to lignocellulose deconstruction using scanning electron microscopy, chemical, and metagenomics approaches. The scanning electron microscopy imagery shows that our consortium can alter the fibers’ organization and conformation, suggesting a possible deconstruction of the lignocellulose. However, it is inappropriate to affirm that the process of deconstruction is effective only by the use of one measurement (i.e., the images of physical alteration of the fibers), as there is no single physical or chemical characteristic of the lignocellulose that can be used to indicate the effectiveness of enzymatic hydrolysis [37]. Moreover, we verified that glucose increased in the medium when the bagasse was exposed to the consortium. Indeed, the reduction in cellulose and hemicellulose content between the 2nd and the 20th weeks of cultivation may support the interpretation. This indicates that not only fibers’ conformation changes but also their chemical composition. Therefore, the consortium was able to deconstruct the lignocellulosic biomass concerning the saccharidic polymers.

We expected that the intensity of the process of deconstruction would not change under a controlled in vitro environment, or both cellulose and hemicellulose would decrease [38,39]. However, our measurements revealed that this was not the case. This finding indicated that an intricate system of interactions was under scrutiny. There was no sign of the lignin’s deconstruction, even though most organisms found in this consortium showed properties enabling them to act as ligninolytic. However, it must be considered that compared to other plant species, such as the eucalyptus, the sugarcane bagasse contains lower amounts of lignin (27.4 and 18%, respectively) [11], suggesting for an order of priority (i.e., more abundant biomass first) of lignocellulosic biomass deconstruction.

Moreover, sequences related to ligninases were found in most genomes comprising the consortiums’ metagenome. We also speculate that the lack of elicitors or other eco-physiological characteristics of the in vitro environment may reduce or absence of the deconstruction of lignin, as previously observed in other cases [40]. Many of the species found (and, among these, some of the most abundant in this consortium) are phylogenetically related to genera known to accomplish this process (e.g., *Pseudomonas*, *Sphingomonas*, *Sphingobium*, *Acinetobacter*, *Variovorax*, *Paenibacillus*, *Pseudoxanthomonas*, and *Chryseobacterium*) [4,16,39,41,42]. Interestingly, *Chryseobacterium* was found to deconstruct lignin in other works [4,39] and is one of the most abundant genera identified in this bacterial consortium (mainly on the 2nd week of cultivation). Conversely, the observation of structures resembling pseudo-lignin droplets, mainly on the 2nd of cultivation, may support a decrease in the ligninases enzymatic activity, since these structures may affect the biomass deconstruction [43], which is indeed observed by the microscopy imagery and chemical analyses from the 20th week of cultivation.

The increase in relative abundance among the most abundant species found in the 2nd week suggests that stochastic processes may also be relevant in the consortium dynamics, i.e., the most relatively abundant species were kept highly abundant in the consortium primarily due to their original high relative abundance. Although it is challenging to prove the influence of stochastic processes in community dynamics, it is broadly recognized that this phenomenon is relevant in bacterial communities and may not be dismissed [38]. In general, the classes found in the consortium presented a highly redundant overall metabolic potential. This may help this consortiums’ engineering efforts when aiming to improve biotechnological interest [4,44,45].

PCA allowed speculation that some taxa may show more in-group similar potential lignocellulosic deconstruction capacities in the consortium. On the other hand, it is essential to consider that the relatively low eigenvalues of both PCAs indicate a weak dimension representation, possibly due to our data’s high dimensionality (quantities of hundreds of different types of sequences, for each of many genomes). This suggests that the decomposition of these data into each family of CAZymes and grouping the MAGs into classes are stringent to this analysis. In summary, we lose relevant information about the DoBL when taxonomically pooling the quantities of sequences, as many sequences indicate the same (or very close) biochemical activity potential over the lignocellulose polymers.

This observation was amended when the sequences were pooled by activity instead of taxonomy and compared each MAG data in overall activities (CAZymes related to ligninases and CAZymes related to hemicellulases and cellulases)—a procedure that was used to build the proposed metabolic model (Figure 10). Nevertheless, the PCA points to each groups’ specificity through the clustering—the taxonomic groups are more similar between themselves in their capacities to deconstruct lignocellulose than to other groups. It is also expected to observe some overlapping between groups, considering that these enzyme gene sequences’ classification schemes are not comprehensive (e.g., in opposition to a taxonomic classification of sequences). Taken together, these results strongly indicate that the consortium shows a taxonomy-defined DoBL to achieve the deconstruction of the lignocellulosic biomass, even though many reactions are shared between the groups. The model shows that, although there is some above-species grouping concerning potential participation in DoBL, this potential is also relevant to species level. Thus, DoBL seems to be species-specific. Although many steps in the deconstruction of the lignocellulosic biomass may be shared among species or above-species groups, at least some steps depend on fewer species for each polymer type in the lignocellulosic biomass.

Showing a broad spectrum of action, GH enzymatic families may catalyze the glycosidic bond’s hydrolysis between carbohydrates [46]. Most MAGs and taxa showed some subfamily members (such GH13) ubiquitously, while other subfamilies were found distributed unevenly, indicating both some overall activities and specific activities. Glycosyl transferases (GTs) are enzymes that catalyze the transfer of saccharide moieties from polysaccharides products in mechanisms of retention or inversion of the substrate [47]. GTs can potentially help deconstruct polysaccharides by depleting the available moieties in the medium, freeing acting enzymes. Many GT families and carbohydrate-binding modules (CBMs), protein modules within enzymes showing a well-defined carbohydrate-binding activity [48], were found in the consortium. CBMs act synergistically, improving the mechanism of action of other enzymes that can act over oligo and polysaccharides. For instance, the CBM2 is a modular enzymatic family found in all Cytophagia and Thermoleophilia, also highly frequent in Actinobacteria classes but less often found in Alphaproteobacteria and Gammaproteobacteria classes, and absent in Bacilli and Oligoflexia classes. This family of enzymes participates in the deconstruction of cellulose and, less often, hemicellulose, assisting the effectiveness of other catalytic regions of the same peptide [49,50].

These observations may indicate the relevance of Cytophagia, Thermoleophilia, and Actinobacteria in the deconstruction of cellulose by this consortium. As a counterexample, CBM50 is a family found in high frequency in all classes of this consortium, showing binding activity to bacterial cell walls, particularly to N-acetylglucosamine residues. CBM50 was found ubiquitous in the consortiums’ MAGs, even though it may not be particularly relevant to the process of deconstruction of the lignocellulose.

Carbohydrate esterases (CEs) catalyze the acylation of substituted saccharides [51]. As CEs act over acylated moieties of polysaccharides, the enzymes promote the lignocellulosic polymers’ deconstruction by allowing other enzymatic families access (such as GHs and GTs) [52]. In particular, CE5 (frequency of 50–100%) shows the activity of hydrolysis of acetylated moieties in polymeric xylan, acetylated xylan, and glucose, a potentially relevant process to the deconstruction of lignocellulose in this consortium. Polysaccharide lyases (PLs) are enzymes that cleave polymers containing uronic acid, resulting in a hexenuronic acid residue and a reducing end [53]. PLs were found sparsely in the consortium’s groups. Bacilli class showed a comparatively elevated frequency of this enzymatic family. Bacteroides class also showed a higher frequency. Gammaproteobacteria and Thermoleophilia showed very low to no sequence of this type of enzyme.

Auxiliary activities (AAs) are redox-active enzymes that may be involved in lignin deconstruction, allowing the GHs, GTs, PLs, and CEs families of enzymes to reach the saccharidic polymers in the biomass [54]. The AA enzymatic family was found in comparatively high frequency in all consortium classes, suggesting that most MAGs can participate in the lignins’ deconstruction.

Overall, the knowledge of each species’ participation in the consortium over the deconstruction of lignocellulose, associated with the knowledge about the potential involvement in other eco-physiological processes, may contribute to the engineering and synthetic biology efforts towards a biotechnologically efficient consortium or controlled steps involved in this process [55,56].

## 5. Conclusions

We recovered a bacterial consortium that is stable mainly in its dynamics on species richness and abundances. The redundancy of the overall metabolism of the groups supports this proposition. Nevertheless, the division of biochemical labor indicated by the sequences related to the deconstruction of lignocellulose suggests that each genome has its particular importance in the consortium structure. Thus, this consortium may contribute to the broadening of the knowledge about the myriad of biochemical processes involved in the deconstruction of lignocellulose and its stability under potential manipulation applications of biotechnological efforts.

## Data Availability

The metagenome assembled genomes (MAGs) and raw sequencing reads have been deposited into GenBank, BioProject PRJNA716287. This Whole Metagenome Shotgun project has been deposited at DDBJ/ENA/GenBank under the accession XXXXXX000000000. The version described in this paper is version XXXXXX010000000.

## Supplementary Materials

The following are available online at https://www.mdpi.com/2076-2607/9/5/995/s1, Figure S1: Scanning electron microscopy of sugarcane fibers used as a sole carbon source in bacterial culture media showing structures resembling pseudo-lignin droplets (yellow arrows), Table S1: Mass balance raw data, Table S2: Sequencing and assembly status of the lignocellulose-decomposing bacterial community, Table S3: Taxonomic classification and genome completeness and status of each MAG obtained from the metagenome binning procedure, Table S4: The number of CAZY domains identified in each MAG found in the consortium.
