# Supplementary material for: Unraveling a Lignocellulose-Decomposing Bacterial Consortium from Soil Associated with Dry Sugarcane Straw by Genomic-Centered Metagenomics
Source: Microorganisms. 2021 May 5;9(5):995. doi: 10.3390/microorganisms9050995 (PMC8170896; doi:10.3390/microorganisms9050995)
Supplement: Supplementary file 1 [file microorganisms-09-00995-s001.zip › microorganisms-1173787-supplementary/TableS2.pdf]

Table S2. Sequencing and assembly of the lignocellulose-decomposing bacterial community.

	Raw Reads	Reads after trimming	Assembled size (bp)	N50 (Kb) / L50	Longest scaffold (bp)	Scaffolds > 300bp
Free: 2nd week	53,311,191	36,795,258	216,621,114	6,524 / 3,706	1,251,422	141,395
Attached: 2nd week	50,362,156	40,708,747	205,005,671	7,330 / 2,355	1,557,200	133,028
Free: 20th week	50,849,621	35,056,982	198,735,922	3,227 / 7,520	1,211,013	156,975
Attached: 20th week	58,663,806	36,039,348	207,655,023	3,510 / 7,599	479,265	161,807
All (megahit)	213,186,774	148,600,335	374,211,453	12,854 / 3,318	1,904,463	127,034
