# Supplementary material for: Unraveling a Lignocellulose-Decomposing Bacterial Consortium from Soil Associated with Dry Sugarcane Straw by Genomic-Centered Metagenomics
Source: Microorganisms. 2021 May 5;9(5):995. doi: 10.3390/microorganisms9050995 (PMC8170896; doi:10.3390/microorganisms9050995)
Supplement: Supplementary file 1 [file microorganisms-09-00995-s001.zip › microorganisms-1173787-supplementary/TableS4.pdf]

Table S4. The number of domains for each MAG found in the consortium. (continue)

Bin	GH	GT	CE	CBM	AA
01	463	209	129	14	77
02	662	323	209	78	75
03	106	77	53	17	39
04	319	156	158	8	76
05	288	183	154	6	48
06	441	179	102	11	46
07	381	245	163	29	54
08	115	112	180	2	67
09	448	444	93	95	61
10	161	155	77	8	52
11	680	194	122	49	33
12	173	189	127	17	70
13	825	281	147	68	51
14	45	132	98	6	75
15	92	122	66	8	27
16	100	285	125	32	45
17	176	171	86	8	89
18	265	201	149	12	137
19	883	202	133	57	33
20	297	120	102	23	54
21	235	124	76	8	83
22	500	162	99	12	45

Table S4. The number of domains for each MAG found in the consortium. (continue)

Bin	GH	GT	CE	CBM	AA
23	668	370	162	65	53
24	141	156	144	9	134
25	499	277	145	68	55
26	888	179	126	42	39
27	89	109	101	5	68
28	721	407	223	95	83
29	896	247	152	90	50
30	120	119	49	8	41
31	845	282	147	52	47
32	319	130	115	12	55
33	885	275	205	111	51
34	161	100	99	7	52
35	159	150	77	13	53
36	663	153	100	41	60
37	575	163	101	59	45
38	244	243	150	22	29
39	542	234	93	69	68
40	385	135	133	12	77
41	1174	311	238	100	41
42	184	169	104	10	119
43	574	160	210	17	71
44	55	107	82	4	60

Table S4. The number of domains for each MAG found in the consortium. (end)

Bin	GH	GT	CE	CBM	AA
45	136	250	112	24	64
46	177	315	78	49	80
47	179	179	142	12	105
48	30	73	83	7	55
49	151	161	107	5	68
50	554	189	149	10	56
51	251	117	108	7	99
52	137	47	88	4	43
