# Supplementary figures and images for: Unraveling a Lignocellulose-Decomposing Bacterial Consortium from Soil Associated with Dry Sugarcane Straw by Genomic-Centered Metagenomics
Source: Microorganisms. 2021 May 5;9(5):995. doi: 10.3390/microorganisms9050995 (PMC8170896; doi:10.3390/microorganisms9050995)

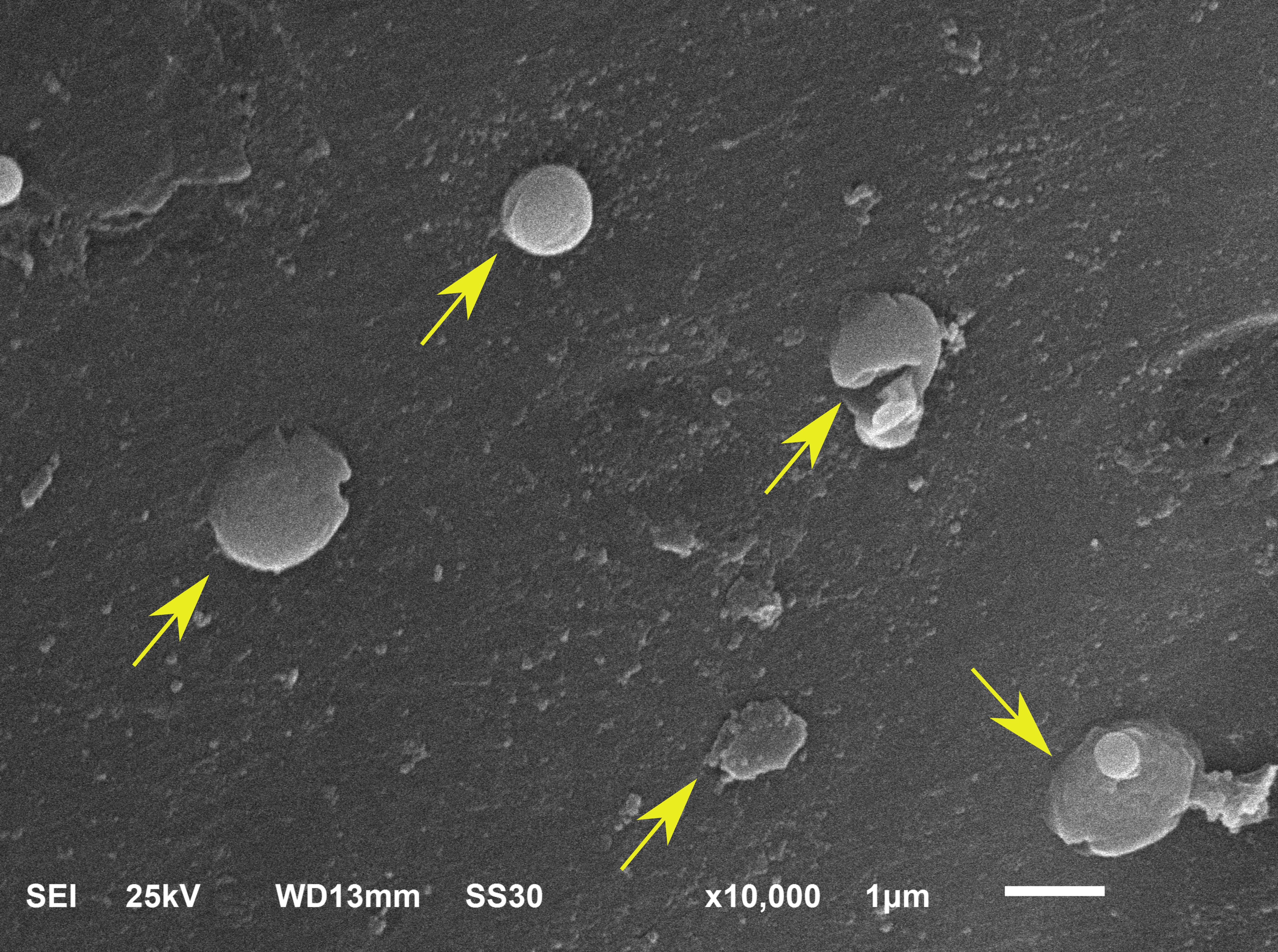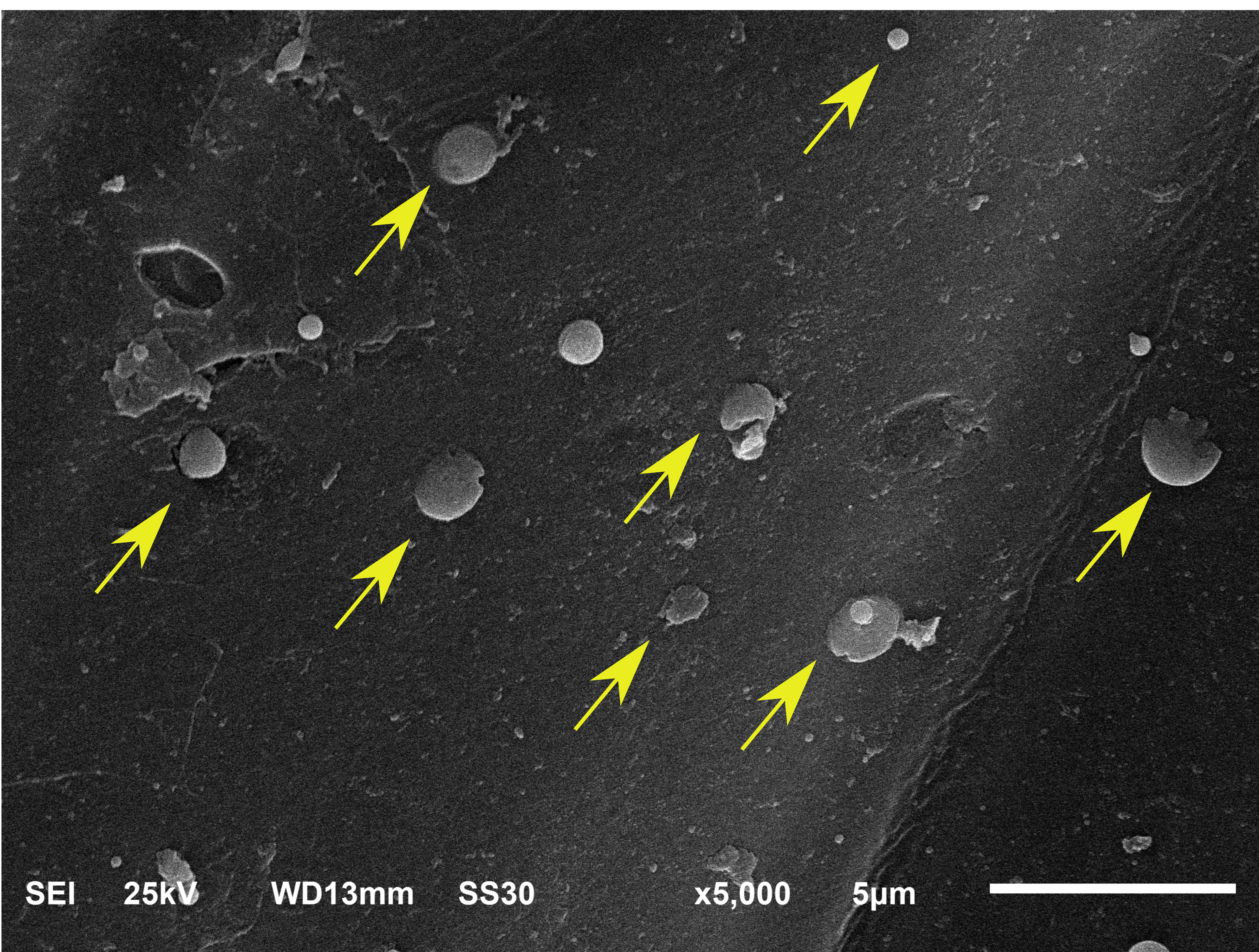

Supplement: Supplementary file 1 [file microorganisms-09-00995-s001.zip › microorganisms-1173787-supplementary/FigureS1.pdf]
